# Association of adverse childhood experiences (ACEs) and substance use disorders (SUDs) in a multi-site safety net healthcare setting

**DOI:** 10.1016/j.abrep.2020.100293

**Published:** 2020-07-03

**Authors:** Daniel J. Bryant, Emil N. Coman, April Joy Damian

**Affiliations:** a Weitzman Institute at Community Health Center, Inc., 19 Grand Street, Middletown, CT 06457, United States; b University of Connecticut Health Disparities Institute, 263 Farmington Ave., MC 7030, Farmington, CT 06030-7030, United States

**Keywords:** Adverse Childhood Experiences (ACEs), Trauma, Substance use disorders, Community health

## Abstract

**Background:**

Adverse childhood experiences (ACEs) and substance use disorders (SUDs) are highly prevalent public health challenges that have been shown to be strongly correlated. Although previous research has suggested a dose-response relationship between ACEs and SUDs, less is known about this phenomenon and the prevalence of ACEs in lower income, racially/ethnically diverse populations. This study sought to examine these relationships in a population treated at a multi-site safety net provider.

**Methods:**

The ACEs survey was delivered as a standard assessment to all behavioral health patients seen at a large Federally Qualified Health Center (FQHC) in Connecticut. 4378 patients completed the questionnaire. Both total score and individual ACE questions were correlated with diagnostic history, according to chi-square and multiple-group structural equation modeling tests.

**Results:**

84.8% of patients reported at least one ACE and 49.1% had an ACE score ≥ 4. Experiencing 1 or more ACEs predicted having any SUD, after controlling for race/ethnicity and gender. Parent substance use, physical abuse, and sexual abuse in particular were the strongest predictors of developing any SUD. Men and non-white individuals were more likely to develop an SUD with lower ACE scores than women and white individuals.

**Conclusions:**

While ACEs predict an increased likelihood of developing any SUD, the nature of this relationship differs by both gender and race/ethnicity. In this FQHC patient population there is no obvious dose-response relationship between ACEs and SUDs. Additional research is required to help understand why the relationship between ACEs and SUDs observed here differs from other populations.

## Highlights

• Adverse childhood experiences are highly correlated with substance use disorders.
• Each individual ACE significantly predicted an SUD.
• In a large sample with high ACEs, no dose-response relationship was found.
• The majority of female patients had an ACE score ≥ 4.
• The prevalence of different ACEs varied significantly by race/ethnicity.

## 1 Introduction

Adverse Childhood Experiences (ACEs) are defined as negative experiences that are potentially traumatic occurring before the age of 18. According to the Centers for Disease Control and Prevention (CDC) 61% of adults in the U.S. have had at least 1 ACE and 16% had 4 or more types of ACEs (Centers for Disease Control and Prevention, 2019). ACEs can take the form of abuse (physical, verbal/emotional, or sexual abuse), neglect (physical and emotional), and household dysfunction (parental incarceration, witnessing domestic violence, parental substance use, parental mental illness, or parental separation/divorce). The impact of ACEs are not limited to childhood; they have lasting impacts on injury (traumatic brain injury, fractures, burns), mental health (depression, anxiety, suicide, PTSD), maternal health (unintended pregnancy, pregnancy complications, fetal death), infectious disease (HIV, STDs), chronic disease (cancer, diabetes), risk behaviors (alcohol and substance use disorder, unsafe sex), and opportunities (education, occupation, income) well into adulthood (Felitti et al., 1998, Leeb et al., 2011).

There is a strong body of research supporting a dose-response relationship between the number of ACEs and the likelihood of developing a substance use disorder (SUD) (Dube et al., 2002, Giano et al., 2019, LeTendre and Reed, 2017, Wu et al., 2010). While connections between childhood adversity and SUDs had been established (Aron, 1975) prior to the seminal ACEs study (Felitti et al., 1998), subsequent studies indicated that ACEs are associated with both early age of substance use initiation (Stein et al., 2017), and greater likelihood of developing an SUD (Dube et al., 2003a). Even within drug using populations, individuals with high ACE scores were more likely to experience negative impacts from substance use such as psychosis secondary to substance use and relapse during treatment (Derefinko et al., 2019, Ding et al., 2014).

Despite the consistent dose response relationship seen in ACEs research (Dube et al., 2003a, Felitti et al., 1998), additional research has shown that the impact of each ACE is not equivalent, especially as it relates to SUDs. Choi and colleagues found that physical abuse, sexual abuse, and parental substance use were all predictors of a lifetime SUD diagnosis while other ACEs were not significant predictors of developing an SUD (Choi, DiNitto, Marti, & Choi, 2017). More specifically, parental substance use was found to be a significant factor in grouping and classifying ACE exposure using latent class analysis and these classes were significant predictors of developing an SUD (Cavanaugh et al., 2015, Shin et al., 2018).

While there is a strong body of research on ACEs and their negative sequelae, there continues to be major gaps in the literature. Notably missing from the literature on ACEs is an analysis of populations with higher average ACE scores defined as 4 or more ACEs (Dube et al., 2003a, Felitti et al., 1998). While studies have found differences between populations, most published studies have shown low to moderate percentages of the population with ACE scores > 4, which subsequently limits the field’s understanding of the health sequalae specifically associated with higher ACEs (Felitti et al., 1998, Guarino et al., 2016, Institute for Safe Families, 2013, Merrick et al., 2018). Additionally, early ACEs research focused on populations that were less diverse, more educated, economically advantaged, and with low average ACE scores (Dube et al., 2002, Edwards et al., 2003, Felitti et al., 1998). Thus, there is a paucity of research examining ACEs among racial/ethnic minority populations and low-income communities. A recent study from Hargreaves and colleagues examined ACEs among low-income predominantly minority residents in the southern United States. Their study found similar distribution of ACEs as previous studies and discovered a higher rate of ED utilization among individuals with higher ACEs among all demographic groups (Hargreaves, Mouton, Liu, Zhou, & Blot, 2019). As a part of the Chicago Longitudinal Study, Mersky, Topitzes, and Reynolds examined ACEs among low-income, exclusively non-white Chicago residents. The study showed similar connections between ACEs and negative health outcomes and only slightly higher ACE prevalence than previous studies (Mersky, Topitzes, & Reynolds, 2013). Thus, there continues to be gaps in the field’s understanding of how high levels of ACEs, poverty, and mental health and substance use interact.

This study had three main objectives: (1) understand the prevalance of ACEs among patients seen in a safety-net, behavioral health setting; (2) examine the association between ACEs and SUDs in a population with high levels of ACEs, including differences based on race/ethnicity and gender; and, (3) explore the specifc relationship between ACEs and SUD beyond the greater than 4 threshold.

## 2 Methods

### 2.1 Sample

The sample consisted of patients enrolled in behavioral healthcare services at Community Health Center, Inc., a federally qualified health center (FQHC) in Connecticut. These patients entered treatment between March 2017 and January 2019. All of these patients were seen by a medical provider prior to being admitted to the behavioral health program. The ACEs data were collected as a part of a semi-structured intake assessment. The intake interview is designed to identify diagnoses, maintain compliance with regulatory bodies such as the local Department of Public Health and the Joint Commission, and help develop a treatment plan. The ACE survey was implemented as a means of improving trauma care for the patient population. The data analyzed was obtained in January 2019. During this time period, 4748 people completed an intake. Of those 4748, every patient who did not have a complete 10 question ACE survey was eliminated unless we could identify answers to the questions elsewhere in the intake. Only one such record was identified where the question of parental divorce was left blank but a note in the intake identified that the patient’s parents had been divorced. Consequently, we successfully obtained completed ACE survey data on 4378 unique individuals (92.2% of eligible population). Our institution review board approved this study as a retrospective of patient data without the need for patient consent.

## 3 Measures

### 3.1 Adverse childhood experiences (ACEs)

The 10 item Adverse Childhood Experiences survey, adapted from the original seven item survey (Felitti et al., 1998), was first used by Dube and colleagues as part of the larger CDC-Kaiser study (Dube, Felitti, Dong, Giles, & Anda, 2003b). The survey asks questions about experiencing during childhood: parental separation, physical neglect, emotional neglect, physical abuse, emotional abuse, sexual abuse, witnessing domestic violence, and household dysfunction defined as parental substance abuse, parental mental illness or suicide attempts, and parental incarceration. These events are scored as a 1 if the individual experienced them (0 if they did not) and summed to reflect the cumulative exposure to ACEs as in previous work in the field (Anda et al., 2010, Dube et al., 2003a, Felitti et al., 1998).

### 3.2 Substance use disorders (SUDs)

Substance use disorder diagnoses were decided by treating clinicians. All clinicians used the DSM-5 criteria to arrive at the diagnosis and any diagnosis, regardless of severity (mild, moderate, or severe) was counted as a positive diagnosis for analyses in this study.

### 3.3 Other covariates

Data on race and ethnicity, gender, and age are collected as a part of routine care at the FQHC and were used in statistical analyses.

## 4 Analysis

We tested differences by gender in categorical outcomes (i.e. each of the 10 binary ACE items) with chi-squares tests, and group differences in continuous outcomes with multiple-group one outcome structural equation models (SEM) (Bollen, 1989, Hayduk, 1987, Wright, 1921) which provide identical results to plain t-tests, but additionally can relax assumptions, e.g., the equality of variances (Coman et al., 2014). The SEM testing simply assesses (with a chi-squared test) whether the model that forces the means of the total ACE scores in males and females, e.g., fits significantly worse than the one where means are estimated as different parameters: if it fits worse, then the equality is not supported by the data. To compare binary outcomes with and without covariates we used logistic regressions of the binary outcome on the grouping of interest, without then with covariates; we adjusted for gender and race/ethnicity. All analyses were performed in Stata 15 (“Stata Statistical Software”, 2017).

## 5 Results

### 5.1 Demographic Characteristics

Table 1 shows the gender, age, race/ethnicity and prevalence of ACEs and SUDs for the sample (N = 4378). The sample was predominantly female (59.5%). The majority of the sample patients did not self-identify as non-Hispanic White (52.6%), with at least 1 out of 5 (21.3%) identifying as Hispanic/Latinx, and 12.2% as non-Hispanic Black. About a third of the sample (33.4%) have been diagnosed with a SUD, and 12.2% having been diagnosed with more than one SUD. The most prevalent forms of SUD were related to tobacco (30.3%), alcohol (14.6%), and opioid use disorder (14.9%).

**Table 1:** Characteristics of Sample [N = 4378].

Characteristic	N	%
Gender		
Male	1773	40.53%
Female	2602	59.47%

Age		
18–19	73	1.67%
20–34	1639	37.44%
35–49	1388	31.70%
50–64	1089	24.87%
65+	189	4.32%

Race/Ethnicity		
Non-Hispanic White	2075	47.40%
Non-Hispanic Black	533	12.17%
Hispanic/Latinx	925	21.13%
Asian	38	0.87%
American Indian/Pacific Islander	15	0.34%
Other	792	18.09%

Substance Use Disorders		
Any SUD	1460	33.35%
>1 SUD	532	12.15%
Alcohol Use Disorder	638	14.57%
Cannabis Use Disorder	349	7.97%
Cocaine Use Disorder	273	6.24%
Hallucinogen Use Disorder	23	0.53%
Inhalant Use Disorder	5	0.11%
Opioid Use Disorder	651	14.87%
Sedative Use Disorder	46	1.05%
Tobacco Use Disorder	1326	30.29%
Other Substance Use Disorder	219	5.00%

### 5.2 Prevalence of ACEs

Table 2a shows the prevalence of individual ACE items by gender. While the mean ACEs score for the sample was 3.7 (*SD* = 2.76), the mean ACEs total for females (*M* = 3.92, *SD* = 2.74) was significantly higher than that of males (*M* = 3.37, *SD* = 2.74). More males reported no ACE than females (16.5% vs. 14.3%, *p* < .01). An overwhelming majority of the sample total (84.8%) reported at least one ACE and almost half of the sample total (49.1%) had an ACE score > 4. Additionally, the majority (52.4%) of the female sample had an ACE score > 4, which was significantly higher than that for males in the sample (44.1%). Among the total sample, similar to other populations (Garland et al., 2019, Lee and Chen, 2017, Stein et al., 2017) the most commonly reported ACEs were parental divorce (57.0%), verbal abuse (47.2%), parental drug use (46.1%), emotional neglect (41.3%), and parental mental illness/suicide (40.9%). Of note, about 1 out 5 (21.0%) patients in the sample reported parental incarceration, and over a quarter of the sample (28.4%) reported witnessing domestic violence. Among males in the sample, the most commonly reported ACEs were parental drug use (45.7%), verbal abuse (44.9%), physical abuse (36.3%), parental mental illness/suicide (36.3%), and emotional neglect (35.8%). Among females in the sample, verbal abuse (48.7%), parental drug use (46.3%), emotional neglect (45.0%), parental mental illness/suicide (44.0%), and sexual abuse (39.6%) were the most commonly reported ACEs. The prevalence of all reported forms of childhood abuse and neglect (verbal abuse, physical abuse, sexual abuse, physical neglect, emotional neglect) and parental mental illness were significantly higher among females than males in the sample size (p < .05).

**Table 2a:** ACE Prevalence by Gender.

	Male		Female		Sample Total	
ACE Total						
0	293	16.53%**	372	14.30%	665	15.19%
1	265	14.95%***	276	10.61%	542	12.38%
2	238	13.42%*	294	11.30%	532	12.15%
3	195	11.00%	296	11.38%	491	11.22%
4+	782	44.11%	1364	52.42%***	2148	49.06%

Specific ACEs						
Verbal Abuse	796	44.90%	1268	48.73%*	2065	47.17%
Physical Abuse	644	36.32%	999	38.39%	1645	37.57%
Sexual Abuse	327	18.44%	1030	39.58%***	1359	31.04%
Emotional Neglect	634	35.76%	1172	45.04%***	1807	41.27%
Physical Neglect	302	17.03%	542	20.83%**	845	19.30%
Parental Drug Use	810	45.69%	1205	46.31%	2017	46.07%
Witnessing Domestic Violence	484	27.30%	757	29.09%	1241	28.35%
Parental Mental Illness/Suicide	644	36.32%	1145	44.00%***	1792	40.93%
Parental Incarceration	365	20.59%	556	21.37%	921	21.04%
Parental Divorce	976	55.05%	1520	58.42%*	2497	57.04%

*Note*.

* *p* < .05.

** *p* < .01.

*** *p* < .001.

Table 2b compares total ACE scores and individual ACEs by race/ethnicity. The total ACE scores differ by race/ethnicity X^2^ (4, *N =* 4378) = 13.8277, *p* < .01) with the highest average total ACE score reported by Hispanic/Latinx (*M* = 4.06, *SD* = 2.81). Six out of the ten ACEs differed significantly by race/ethnicity. While non-Hispanic whites had the highest prevalence witnessing domestic violence (43.4%), non-Hispanic blacks had the highest prevalence of physical abuse (36.6%), physical neglect (50.5%), parental mental illness/suicide (34.2%), parental incarceration (64.7%), and parental divorce (46.9%).

**Table 2b:** ACE Individual Symptoms Prevalence by Race/Ethnicity of Patients.

	Non-Hispanic White	Non-Hispanic Black	Hispanic/Latinx	Other	Unknown	Sample Total
ACE Total						
0	14.8%	12.8%	16.3%	18.3%	15.8%	15.2%
1	12.9%	10.5%	11.4%	12.0%	14.0%	12.4%
2	12.4%	9.8%	12.7%	13.2%	12.1%	12.2%
3	10.9%	12.8%	10.8%	12.4%	10.9%	11.2%
4+	49.0%	54.2%	48.9%	44.2%	47.1%	49.1%

Specific ACEs						
Verbal abuse	38.3%	37.5%	38.3%	34.3%	35.5%	37.6%
Physical Abuse	29.1%	36.6%***	34.2%	27.5%	29.6%	31.0%
Sexual Abuse	40.5%	44.8%	41.2%	39.8%	41.4%	41.3%
Emotional Neglect	18.1%	21.4%	20.9%	17.9%	19.7%	19.3%
Physical Neglect	48.9%	50.5%***	41.4%	41.0%	41.8%	46.1%
Parental Drug use	27.9%	33.6%	28.1%	26.7%	26.4%	28.4%
Witnessing DV	43.4%*	36.0%	38.5%	40.2%	40.7%	40.9%
Parental MI/Suicide	17.3%	34.2%***	22.7%	18.7%	20.9%	21.0%
Parental Incarceration	55.7%	64.7%***	58.6%	52.6%	54.5%	57.1%
Parental Divorce	49.8%	46.9%*	44.4%	43.4%	43.9%	47.2%

*Note*. **p < .01.

* *p* < .05.

*** *p* < .001.

### 5.3 Substance use disorder likelihood as a result of ACE symptoms

Table 3a shows for each gender and racial/ethnic group the odds ratios of having any SUD (no SUD being the reference category) if patients reported 1, 2, 3, or 4 (or more) ACE symptoms. Male patients are more likely to have an SUD if they experienced either 1, 2, 3, or >4 ACE symptoms (*OR* = 1.41, *OR* = 1.38, *OR* = 1.27, and *OR* = 1.22, respectively) whereas female patients are more likely to have a SUD if they experienced 3, or 4 (or more) ACEs (*OR* = 1.18 and *OR* = 1.22, respectively). The odds of having an SUD increase in non-Hispanic White patients only if they reported 4 (or more) ACE symptoms, whereas non-Hispanic Black patients are more likely to have an SUD if they experienced 2, or 4 (or more) ACEs (*OR* = 1.67 and *OR* = 1.22, respectively), and Hispanic patients are more likely to have a SUD if they experienced 2, 3, or 4 (or more) ACEs (*OR* = 1.49, *OR* = 1.57, and *OR* = 1.36, respectively).

**Table 3a:** Odds Ratios of Having Any SUD if Patients Reported 1, 2, 3, or 4 and More ACE Symptoms, by Gender and Race/Ethnicity with 95% Confidence Intervals.

	Male	Female	Non-Hispanic White	Non-Hispanic Black	Hispanic/Latinx	Other	Unknown	All
0 ACEs (reference)	1.0	1.0	1.0	1.0	1.0	1.0	1.0	1.0
1 ACE	1.41* [1.00–**1.99]**	1.11 [0.73–1.69]	1.04 [0.73–1.47]	1.81 [0.82–4.02]	1.75 [0.83–3.69]	1.64 [0.43–6.24]	1.58 [0.81–3.06]	1.33* [1.03–**1.72]**
2 ACEs	1.38*** [1.15–**1.64]**	1.15 [0.94–1.41]	1.14 [0.96–1.36]	1.67** [1.13–**2.49]**	1.49* [1.05–**2.12]**	1.62 [0.88–2.98]	1.16 [0.82–1.64]	1.26*** [1.11–**1.43]**
3 ACEs	1.27*** [1.12–**1.44]**	1.18** [1.04–**1.34]**	1.12 [0.99–1.26]	1.19 [0.92–1.54]	1.57*** [1.25–**1.97]**	**1.57** **[1.06**–**2.34]**	0.99 [0.77–1.28]	1.19*** [1.09–**1.30]**
4 + ACEs	1.22*** [1.13–**1.30]**	1.22*** [1.13–**1.31]**	1.16*** [1.09–**1.24]**	1.22** [1.04–**1.42]**	1.36*** [1.18–**1.56]**	1.24 [0.96–1.61]	1.06 [0.92–1.21]	1.18*** [1.12–**1.24]**

*Note*.

* *p* < .05.

** *p* < .01.

*** *p* < .001.

Table 3b reports the change in likelihood of having an SUD when patients report individual ACEs, controlling for sociodemographics (e.g., gender and race/ethnicity). Each individual ACEs predicted having any SUD, and they each predicted at least one specific SUD. We found for example significantly higher odds for alcohol use disorder for individuals who experienced in their childhood either: physical abuse (*OR* = 1.39), or physical neglect (*OR* = 1.29), or emotional neglect (*OR* = 1.31), or parental drug use (1.77), or witnessing domestic violence (*OR* = 1.34). The odds of having a cannabis use disorder were higher for patients who experienced any of the individual ACEs (all ORs were significant, see Table 3b), while the odds of having a cocaine use disorder were higher for patients who experienced emotional neglect (*OR* = 1.38), physical abuse (*OR* = 1.51), sexual abuse (*OR* = 1.67), or parental drug use (*OR* = 1.80). Finally, the odds of having an opioid use disorder were higher for patients who experienced physical abuse (*OR* = 1.29), sexual abuse (*OR* = 1.32), parental drug use (*OR* = 1.65) or parental incarceration (*OR* = 1.30).

**Table 3b:** Adjusted Odds Ratios with 95% CI for Specific SUD by Specific Adverse Childhood Experiences with 95% Confidence Intervals.

	Verbal Abuse	Physical Abuse	Sexual Abuse	Emotional Neglect	Physical Neglect	Parental Drug Use	Witnessing DV	Parental MI/Suicide	Parental Incarceration	Parental Divorce
Any SUD	1.41*** [1.23–**1.60]**	1.51*** [1.32–**1.73]**	1.47*** [1.27–**1.70]**	1.32*** [1.15–**1.51]**	1.45*** [1.23–**1.70]**	2.07*** [1.81–**2.37]**	1.36*** [1.18–**1.57]**	1.28*** [1.12–**1.47]**	1.42*** [1.21–**1.67]**	1.30*** [1.14–**1.48]**
>1 SUD	1.33*** [1.10–**1.59]**	1.39*** [1.16–**1.68]**	1.57*** [1.28–**1.93]**	1.17 [0.97–1.41]	1.17 [0.92–1.47]	1.90*** [1.57–**2.29]**	1.31*** [1.08–**1.60]**	1.16 [0.92–1.31]	1.24 [0.99–1.55]	1.12 [0.93–1.35]
Alcohol Use Disorder	1.18 [0.99–1.40]	1.39*** [1.17–**1.65]**	1.20 [0.99–1.45]	1.29** [1.08–**1.54]**	1.31* [1.06–**1.62]**	1.77*** [1.48–**2.10]**	1.34** [1.11–**1.61]**	1.10 [0.92–1.31]	1.10 [0.89–1.35]	1.07 [0.90–1.27]
Cannabis Use Disorder	1.74*** [1.39–**2.18]**	1.40** [1.12–**1.74]**	1.62*** [1.28–**2.18]**	1.50*** [1.20–**1.88]**	1.42** [1.10–**1.84]**	1.79*** [1.43–**2.24]**	1.38** [1.09–**1.74]**	1.59*** [1.27–**1.98]**	1.67*** [1.30–**2.13]**	1.37** [1.09–**1.72]**
Cocaine Use Disorder	1.38*** [1.08–**1.77]**	1.51*** [1.18–**1.93]**	1.67*** [1.28–**2.18]**	1.24 [0.97–1.60]	1.24 [0.92–1.67]	1.80*** [1.40–**2.31]**	1.23 [0.94–1.60]	1.08 [0.84–1.39]	1.11 [0.83–1.50]	1.01 [0.79–1.30]
Opioid Use Disorder	1.15 [0.97–1.37]	1.29** [1.09–**1.54]**	1.32** [1.09–**1.59]**	1.02 [0.86–1.22]	1.15 [0.93–1.43]	1.65*** [1.40–**1.97]**	1.14 [0.94–1.37]	1.07 [0.90–1.27]	1.30* [1.06–**1.60]**	1.17 [0.98–1.39]

*Note*.

* *p* < .05.

** *p* < .01.

*** *p* < .001.

## 6 Discussion and conclusion

Our data showed positive associations between ACEs and SUDs, which is consistent with previous findings. However, our study also suggested several new findings. While ACEs were associated with greater likelihood of developing an SUD, no obvious dose-response relationship was observed between ACEs and SUDs. There are multiple potential explanations for this finding. While studies have shown a dose-response relationship for this correlation (Dube et al., 2003a, Felitti et al., 1998), no previous studies had comparable rates of ACEs, which may change the dose-response relationship observed in previous studies. Additionally, protective factors such as resiliency, which were not measured in this study, could explain this observation. More specifically, help seeking behavior is demonstrated by this entire patient sample as they were all assessed for ACEs while seeking behavioral health care, which may demonstrate their resilience or be a confounding variable on its own.

Similar to other studies, parental drug use was the strongest predictor of any SUD as well as all specific SUDs we evaluated. Previous studies found that other specific ACEs impacted men and women differently in terms of the development of SUDs (Choi, DiNitto, Marti, & Choi, 2017). Our study found physical abuse to be the second strongest predictor of developing any SUD whereas sexual abuse was the second strongest predictor for all specific SUDs with the exception of alcohol use disorder. In fact, sexual abuse was not a significant predictor of alcohol use disorder at all. This is in contrast to other studies that have shown greater variation in the impact of specific ACEs and higher odds ratios compared with our sample (Dube et al., 2003b).

### 6.1 Implications, limitations, and strengths

There are several limitations to this study that are worth noting. The main limitation of this study is the selection bias. Nearly 95,000 patients treated in primary care at the FQHC but not seeking behavioral healthcare are not reflected in this sample, thereby limiting the generalizability of the study’s findings. The diagnosis data is also a potential limitation as these diagnoses were derived by trained clinicians using the DSM 5 criteria, but without using any structured instruments to assess the accuracy of their diagnoses. For example, some clinicians may have opted for less severe diagnoses, such as an adjustment disorder, for fear of adding stigma or may only have included diagnoses on the problem list that the patient wanted included in their care plan.

Despite these limitations, this study shows higher ACE scores in this population than any previously published study the authors were able to find. For example, whereas only 6.2% of participants in the original study had an ACE score of 4 or higher, 49% of our population had scores ≥ 4. Furthermore, our sample size was approximately 10 times that of the only other study which demonstrated similar levels of ACEs (Guarino et al., 2016). Additionally, our study included a more diverse sample than the original study where 79.8% of participants were white compared to only 47.4% of this sample (Felitti et al., 1998). Larger scale studies such as the Behavioral Risk Factor Surveillance Survey (BRFSS) have contained even less diverse sampling with 82.9% of respondents being non-Hispanic white (Lee & Chen, 2017). Additionally, our sample also has significantly higher ACE scores. The Connecticut 2017 BRFSS examined 8 of the 10 ACEs as a part of that year’s screening. Connecticut’s BRFSS data showed that Connecticut residents had nearly half as many ACEs as our sample. While in our sample 57% of patients parents were divorced or separated only 26.2% of respondents in the BRFSS data had separated parents. While 27.9% of BRFSS respondents had experienced verbal abuse as children whereas 47.7% of our sample endorsed this ACE. Connecticut’s sample showed 9.4% of respondents had experienced sexual assault as a child while three times as many (31%) in our sample had experienced sexual assault in childhood. The Connecticut data did not separate respondents with 4 or more ACEs, but only 7.2% had 5 or more compared with 38.7% of our sample. In every domain our sample experienced more adversity than large scale sampling in the same state (Connecticut Department of Public Health, 2018).

This study also offers multiple pathways for additional research. While the study shows connections between both total and individual ACEs and SUDs, the impact is much lower than previous studies. Understanding the connection between increased ACE scores and reduced risk of developing an SUD as compared to those with lower ACEs could offer important understanding of resiliency and risk taking behavior. While this study does not show a dose response relationship between ACEs and SUD, additional research might show that additional ACEs have a negative impact on the trajectory of recovery for individuals with SUDs and higher ACE scores. Lastly, research into whether there is an absence of a dose-response relationship with other health consequences linked to ACEs including mental health diagnoses, chronic medical conditions, and early death are potential research questions to examine with this sample as part of a larger effort to better promote the health and well-being of historically underserved and understudied populations.

## CRediT authorship contribution statement

**Daniel J. Bryant:** Conceptualization, Data curation, Investigation, Methodology, Writing - original draft, Writing - review & editing. **Emil N. Coman:** Conceptualization, Methodology, Formal analysis, Visualization, Writing - original draft. **April Joy Damian:** Conceptualization, Supervision, Writing - original draft, Writing - review & editing.

## Declaration of Competing Interest

The authors declared that there is no conflict of interest.
